# Microplastic ingestion affects the lateralised processing of predator stimuli in fish

**DOI:** 10.1111/jfb.70169

**Published:** 2025-08-06

**Authors:** Georgiana Andrei, Alessandro Colombani, Elia Gatto, Marco Scoponi, Luigi Abelli, Annalaura Mancia, Cristiano Bertolucci, Tyrone Lucon‐Xiccato

**Affiliations:** ^1^ Department of Life Sciences and Biotechnology University of Ferrara Ferrara Italy; ^2^ Department of Chemical, Pharmaceutical and Agricultural Sciences University of Ferrara Ferrara Italy; ^3^ Istituto sulla Sintesi Organica e Fotoreattività del CNR, Research unit at the Department of Life Sciences and Biotechnology University of Ferrara Ferrara Italy; ^4^ Department of Biology University of North Florida Florida USA

**Keywords:** cognitive ecology, cognitive plasticity, environmental pollution, laterality

## Abstract

Microplastic ingestion affects fish brains at the molecular level, but its impact on cognitive phenotype remains unclear. We fed zebrafish (*Danio rerio*) food containing either polyethylene or poly(butylene‐adipate‐co‐terephthalate) microplastics for 20 days and assessed their lateralisation, which reflects how information processing is split between brain hemispheres. No changes appeared in rotational or mirror tests, but lateralisation was disrupted in the detour test when facing a predator model. These results suggest microplastic ingestion can impair specific cognitive traits at the phenotypic level.

Microplastics, plastic fragments smaller than 5 mm, have become one of the most pervasive pollutants in natural habitats (Rillig et al., 2017; Scheurer & Bigalke, 2018), including aquatic ecosystems (Li et al., 2020; Lindeque et al., 2020). When ingested by fish, they induce several toxic effects, such as inflammation (Kim et al., 2021; Lu et al., 2016; Qiao et al., 2019) and alterations in physiology (Sharifinia et al., 2020; Wang et al., 2019), metabolism (Boopathi et al., 2023; Cedervall et al., 2012) and other related biological processes (Huang et al., 2022). Although the typical size of microplastics may prevent direct penetration into the brain, the resulting physiological disruptions are expected to indirectly affect brain function (Maille & Schradin, 2017). Moreover, it has recently been shown that microplastics may affect blood flow to the brain (Huang et al., 2025). Accordingly, various studies have reported changes in gene expression and neurotransmitter levels in the brains of fish exposed to microplastics (Barboza et al., 2018, 2023; Ding et al., 2023; Hoyo‐Alvarez et al., 2022; Huang et al., 2023). However, studies investigating the cognitive phenotype found no significant effects on learning, cognitive flexibility and inhibitory control (Irwin et al., 2024; Lucon‐Xiccato et al., 2025). In this study, we investigated whether microplastics could lead to phenotypic effects on a fundamental cognitive function: lateralisation, defined as the division of information processing between the two cerebral hemispheres (Bisazza et al., 1998).

Following the treatment procedure by Lucon‐Xiccato et al. (2025), two groups of zebrafish (*Danio rerio*) were fed commercial dry food mixed with polyethylene (PE; *N* = 35) or poly(butylene‐adipate‐co‐terephthalate) (PBAT; *N* = 35) microplastics (0.01 g of food and 100 μg of microplastics per fish/day) for 20 days. PE was selected as a common conventional plastic and PBAT as a common biodegradable plastic. A control group (*N* = 35) received only dry food. Fish were kept in groups of five in 40 × 30 × 22 cm tanks under standard conditions (28 ± 1°C, 14 h:10 h light–dark cycle).

After the treatment, subjects' lateralisation was assessed using a battery of three standard tests based on asymmetric behaviours: the rotational test, the mirror test and the detour test. In the rotational test (De Russi et al., 2025; Rovegno et al., 2025), each subject was individually tested in a circular white tank to observe its spontaneous swimming behaviour. Immediately after this, the subject was transferred to an octagonal tank with mirrored walls to assess visual lateralisation in response to their own reflection, which simulates a conspecific (Lucon‐Xiccato et al., 2020; Rovegno et al., 2025). Both the rotational and mirror tests lasted 20 min. The trials were recorded using a Sony Handycam HDR‐CX405 (resolution camera 1920 × 1080, 25 frames per second) located approximately 50 cm above the tanks. Using the recordings and a custom stopwatch software (Ciclic Timer, written in Delphi 5 Borland), we calculated the time each subject spent swimming both clockwise and counterclockwise close to the tank edge (within two body lengths, 3 cm) in the rotational test as a measure of lateralisation in motor functions. In the mirror test, we similarly measured the time spent swimming clockwise and counterclockwise near the mirror (within one body length). Clockwise swimming in the mirror test is assumed to reflect left‐eye use and, due to the decussation of the optic nerves, right‐hemisphere processing of the social stimulus, and vice versa (Sovrano et al., 1999; De Santi et al., 2001). Finally, subjects were tested in a detour test to evaluate lateralisation in eye preference when inspecting a predator model. We used the predator model because a recent study suggested that, under certain conditions, the detour test using non‐biological stimuli may not be reliable in fish (Roche et al., 2020). However, research conducted on our study species using our specific stimulus has not raised such concerns and has demonstrated substantial within‐ and between‐study reliability (Bisazza et al., 2007; Facchin et al., 2009). Following Rovegno et al. (2025), each subject was gently guided through a corridor toward a plastic predator dummy placed behind a barrier made of vertical sticks. The dummy‐predator consisted of a fish lure (18 cm) resembling a natural predator of zebrafish, the Indian leaf fish, *Nandus nandus* (Parichy & Postlethwait, 2020). The direction of the turn made in front of the barrier (left or right) was used to infer hemisphere use and thus lateralisation in response to the predator stimulus (Sovrano et.al, 1999; De Santi et al., 2001): a right turn corresponded to left‐eye use (right hemisphere processing) and a left turn indicated right‐eye use (left hemisphere processing). We collected 10 consecutive detour trials for each subject.

For all the tests, we computed two lateralisation indices. The relative lateralisation index indicated the average directionality of swimming or turning preference of each subject. It ranged from −1 to +1, where −1 indicates a counterclockwise swimming preference (in the rotational and mirror tests) or a left turning preference (in the detour test), and +1 indicates a clockwise swimming preference (rotational and mirror tests) or a right turning preference (detour test). For the rotational and the mirror test, the relative lateralisation index was computed as: (time spent swimming in the right direction − time spent swimming in the left direction)/(time spent swimming in the right direction + time spent swimming in the left direction). For the detour test, the relative lateralisation index was computed as: (number of turns right − numbers of turns left)/total number of turns. The second index is the absolute lateralisation index, which is the absolute value of the relative lateralisation index and describes the strength of lateralisation, regardless of its direction (De Russi et al., 2025).

A one‐way analysis of variance (ANOVA) with treatment as fixed effect revealed no significant effect of microplastics on the relative (*F*_2,99_ = 1.160, *p* = 0.566) and the absolute (*F*_2,99_ = 1.387, *p* = 0.255) lateralisation index of the rotational test (Figure 1d). Also in the mirror test, the ANOVA did not show a significant effect of the treatment (relative index: *F*_2,88_ = 0.015, *p* = 0.985; absolute index: *F*_2,88_ = 0.755, *p* = 0.473; Figure 1e).

**FIGURE 1:**
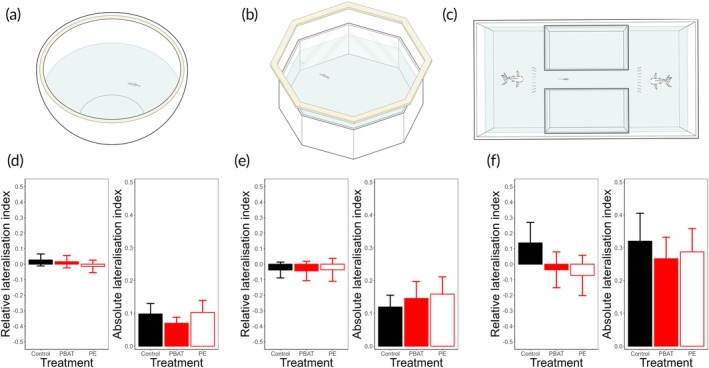
Apparatuses used in the (a) rotational test, (b) mirror test and (c) detour test. Means and standard errors of the relative lateralisation index and the absolute lateralisation index of the subjects assessed in the (d) rotational test, (e) mirror test and (f) detour test. PBAT, poly(butylene‐adipate‐co‐terephthalate); PE, polyethylene.

In the detour test, a generalized linear‐mixed effects model on the outcome of each trial (0 = left turn, 1 = right turn) did not show a significant effect of the trial number (χ^2^_1_ = 0.929, interaction trial × treatment *χ* ^2^_2_ = 0.914), suggesting that fish did not alter their behaviour across the 10 trials. An ANOVA revealed a significant effect of treatment (Figure 1f) on the relative lateralisation index (*F*_2,99_ = 3.321, *p* = 0.040), but not on the absolute lateralisation index (*F*_2,99_ = 0.545, *p* = 0.582). The post hoc tests showed a significant difference in the relative lateralisation index between the control and the PE group (*p* = 0.048), but there was no significant difference between the control and the PBAT group (*p* = 0.122) and between the PE and PBAT groups (*p* = 0.912). A one‐sample *t*‐test revealed that the relative index of the control group was significantly greater than zero, indicating a preference to observe the dummy predator with the left eye, thus processing it with the right hemisphere (*t*_34_ = 2.111, *p* = 0.042). Conversely, the subjects exposed to microplastics did not show a viewing preference for the predator stimulus and thus no lateralisation (PE: *t*_32_ = −1.139, *p* = 0.263; PBAT: *t*_32_ = −0.641, *p* = 0.526).

Our findings suggest that microplastic ingestion does not affect the lateralisation of cognitive functions involved in the rotational and mirror tests. These functions likely play a role in determining spontaneous locomotion direction and the visual processing of conspecifics. However, we cannot exclude the possibility that the absence of an effect was due to these functions not being lateralised in the studied population. Indeed, we did not observe any significant population bias in the control group. In contrast, the processing of a predator stimulus in the detour test was lateralised in the control population. For this function, our analysis revealed a significant disruption due to the microplastic treatment. Specifically, fish in both the PE‐ and PBAT‐treated groups were not lateralised, unlike those in the control group.

Considering also previous studies, we conclude that although microplastic ingestion appears not to affect learning, cognitive flexibility or inhibitory control (Irwin et al., 2024; Lucon‐Xiccato et al., 2025), it does influence certain aspects of cerebral lateralisation. This may be because lateralisation represents a more fundamental aspect of brain function and may therefore be more susceptible to molecular alterations in the brain. Notably, the effects of microplastics were similar regardless of their origin. This raises concerns about biodegradable plastics (e.g. PBAT), which may generate low‐molecular‐weight polymer chains more rapidly than conventional plastics, potentially leading to greater impacts on aquatic organisms.

What does altered lateralisation mean for fish exposed to microplastics in natural environments? It is generally believed that lateralisation in a population results from both genetic and plasticity adaptations (reviewed in Bisazza & Lucon‐Xiccato, 2025). In particular, fish from high‐predator environments tend to exhibit stronger lateralisation, which enhances predator avoidance and survival (Brown et al., 2004; Ferrari et al., 2015). Disruptions in lateralisation due to microplastic exposure could lead to a phenotype‐environment mismatch, potentially reducing fitness. Additionally, microplastics may not be the only anthropogenic factor affecting lateralisation in fish populations. Water acidification (Domenici et al., 2012), hypoxia (Lucon‐Xiccato et al., 2014), rising temperatures (Domenici et al., 2014), artificial illumination (De Russi et al., 2025) and various chemical pollutants (Merola et al., 2024; Suriyampola et al., 2024) have all been observed to alter lateralisation. Consequently, the effects due to microplastics could be additive to those caused by other environmental stressors, potentially leading to even more pronounced fitness consequences.

## AUTHOR CONTRIBUTIONS

G.A.: Investigation, formal analysis, data curation, writing – original draft, visualization. A.C.: Investigation. E.G.: Methodology, writing – review and editing; M.S.: Resources, writing – review & editing. L.A.: Writing – review and editing. A.M.: Writing – review and editing. C.B.: Conceptualization, writing – review and editing, funding acquisition. T.L.‐X.: Conceptualization, methodology, writing – original draft, supervision, funding acquisition.
